# Identifying CpG sites associated with eczema via random forest screening of epigenome-scale DNA methylation

**DOI:** 10.1186/s13148-015-0108-y

**Published:** 2015-07-21

**Authors:** B. M. Quraishi, H. Zhang, T. M. Everson, M. Ray, G. A. Lockett, J. W. Holloway, S. R. Tetali, S. H. Arshad, A. Kaushal, F. I. Rezwan, W. Karmaus

**Affiliations:** Division of Epidemiology, Biostatistics, and Environmental Health, School of Public Health, University of Memphis, 236A Robison Hall, Memphis, TN 38152 USA; Department of Epidemiology, and Biostatistics, Arnold School of Public Health, University of South Carolina, 800 Sumter Street, Columbia, SC 29208 USA; Human Development and Health, Faculty of Medicine, University of Southampton, Southampton General Hospital, Southampton, SO16 6YD UK; Clinical and Experimental Sciences, Faculty of Medicine, University of Southampton, Southampton General Hospital, Southampton, SO16 6YD UK; The David Hide Asthma and Allergy Research Centre, St Mary’s Hospital, Parkhurst Road, Newport, Isle of Wight, PO30 5TG UK; Human Development and Health, Faculty of Medicine, University of Southampton, University Road, Southampton, SO17 1BJ UK

**Keywords:** Eczema, Allergic disease, DNA methylation, Epigenome-scale, Epigenetics, Random forest, F_1_ and F_2_ generations, CpG

## Abstract

**Background:**

The prevalence of eczema is increasing in industrialized nations. Limited evidence has shown the association of DNA methylation (DNA-M) with eczema. We explored this association at the epigenome-scale to better understand the role of DNA-M.

Data from the first generation (F_1_) of the Isle of Wight (IoW) birth cohort participants and the second generation (F_2_) were examined in our study. Epigenome-scale DNA methylation of F_1_ at age 18 years and F_2_ in cord blood was measured using the Illumina Infinium HumanMethylation450 Beadchip. A total of 307,357 cytosine-phosphate-guanine sites (CpGs) in the F_1_ generation were screened via recursive random forest (RF) for their potential association with eczema at age 18. Functional enrichment and pathway analysis of resulting genes were carried out using DAVID gene functional classification tool. Log-linear models were performed in F_1_ to corroborate the identified CpGs. Findings in F_1_ were further replicated in F_2_.

**Results:**

The recursive RF yielded 140 CpGs, 88 of which showed statistically significant associations with eczema at age 18, corroborated by log-linear models after controlling for false discovery rate (FDR) of 0.05. These CpGs were enriched among many biological pathways, including pathways related to creating transcriptional variety and pathways mechanistically linked to eczema such as cadherins, cell adhesion, gap junctions, tight junctions, melanogenesis, and apoptosis. In the F_2_ generation, about half of the 83 CpGs identified in F_1_ showed the same direction of association with eczema risk as in F_1_, of which two CpGs were significantly associated with eczema risk, cg04850479 of the *PROZ* gene (risk ratio (RR) = 15.1 in F_1_, 95 % confidence interval (CI) 1.71, 79.5; RR = 6.82 in F_2_, 95 % CI 1.52, 30.62) and cg01427769 of the *NEU1* gene (RR = 0.13 in F_1_, 95 % CI 0.03, 0.46; RR = 0.09 in F_2_, 95 % CI 0.03, 0.36).

**Conclusions:**

Via epigenome-scaled analyses using recursive RF followed by log-linear models, we identified 88 CpGs associated with eczema in F_1_, of which 41 were replicated in F_2_. Several identified CpGs are located within genes in biological pathways relating to skin barrier integrity, which is central to the pathogenesis of eczema. Novel genes associated with eczema risk were identified (e.g., the *PROZ* and *NEU1* genes).

**Electronic supplementary material:**

The online version of this article (doi:10.1186/s13148-015-0108-y) contains supplementary material, which is available to authorized users.

## Background

The increasing prevalence of allergic conditions including eczema is a major public health concern in industrialized nations [1]. The prevalence of eczema is reported to be 10–30 % in children and 1–3 % in adults of the developed world [2]. In addition to the physical discomfort to the affected individual and the social burden on their families, eczema has a huge economic impact on nations’ health care budgets [3].

Eczema is a chronic condition involving a complex interplay of genetic, epigenetic, and environmental factors [4–6]. So far, DNA methylation (DNA-M) remains the most studied mechanism with potential answers to epigenetic regulation of gene function [7, 8]. The Illumina Infinium HumanMethylation450 Beadchip has the ability to measure DNA methylation at more than 450 K cytosine-phosphate-guanine sites (CpGs), which provides rich information for various epigenetic studies. Epigenome-scale studies offer an immense opportunity to understand disease pathophysiology, but there are also concerns about the challenges associated with this type of studies. A recent review published in 2014 by Paul et al. highlighted the potential challenges in the field of epigenomics [9] such as study design, methodologies of obtaining biologic samples, high dimensionality, and highly correlated data [9, 10].

Random forest (RF) is a machine learning algorithm used for classification and has the ability to efficiently handle high dimensionality and highly correlated data [11]. The R package was used in this study to screen CpG sites potentially associated with eczema. RF is composed of classification trees with each tree constructed using randomly selected bootstrap samples. Misclassification rates calculated based on testing samples can be used to estimate the accuracy of the forests.

In this study, we utilized a method built upon RF to screen specific CpGs potentially associated with eczema using data in the first generation (F_1_) at age 18 years and functionally annotated the genes of the identified CpGs using DAVID [12] to understand the biological pathways. For the identified CpGs via the RF-based method, we further examined their statistical significance on their linear association with eczema risk at age 18 years using log-linear models and replicated the findings from the F_1_ in the second generation (F_2_).

## Results

Eczema frequencies in F_1_ (18 years) and in F_2_ (3, 6, and 12 months) generations stratified by sex indicated that females had higher eczema prevalence than males at 18 years of age in the F_1_ generation, and the prevalence switched in the newborns of the F_2_ generation (Table 1). This is consistent with the gender-reversal pattern of eczema reported in our earlier work [13].

**Table 1:** Eczema status in male and female cohort participants in the F_1_ and F_2_ generations (chi-square tests)

F_1_ generation				
Independent variables		Females	Males	Chi-square
		(*n* = 244)	(*n* = 122)	*P* value
Eczema status	Yes	37 (15.2 %)	9 (7.3 %)	0.051
	No	207 (84.8 %)	113 (92.6 %)	
F_2_ generation				
Independent variables		Boys	Girls	Chi-square
		(*n* = 60)	(*n* = 56)	*P* value
Age 3 months	Yes	9 (15.0 %)	2 (3.6 %)	0.048
Eczema status	No	44 (73.3 %)	53 (94.6 %)	
	Missing	7 (11.7 %)	1 (1.8 %)	
Age 6 months	Yes	13 (21.7 %)	6 (10.7 %)	0.162
Eczema status	No	39 (65.0 %)	43 (76.8 %)	
	Missing	8 (13.3 %)	7 (12.5 %)	
Age 12 months	Yes	9 (15.0 %)	5 (8.9 %)	0.521
Eczema status	No	37 (61.7 %)	36 (64.3 %)	
	Missing	14 (23.3 %)	15 (26.8 %)	

**Fig. 1:**
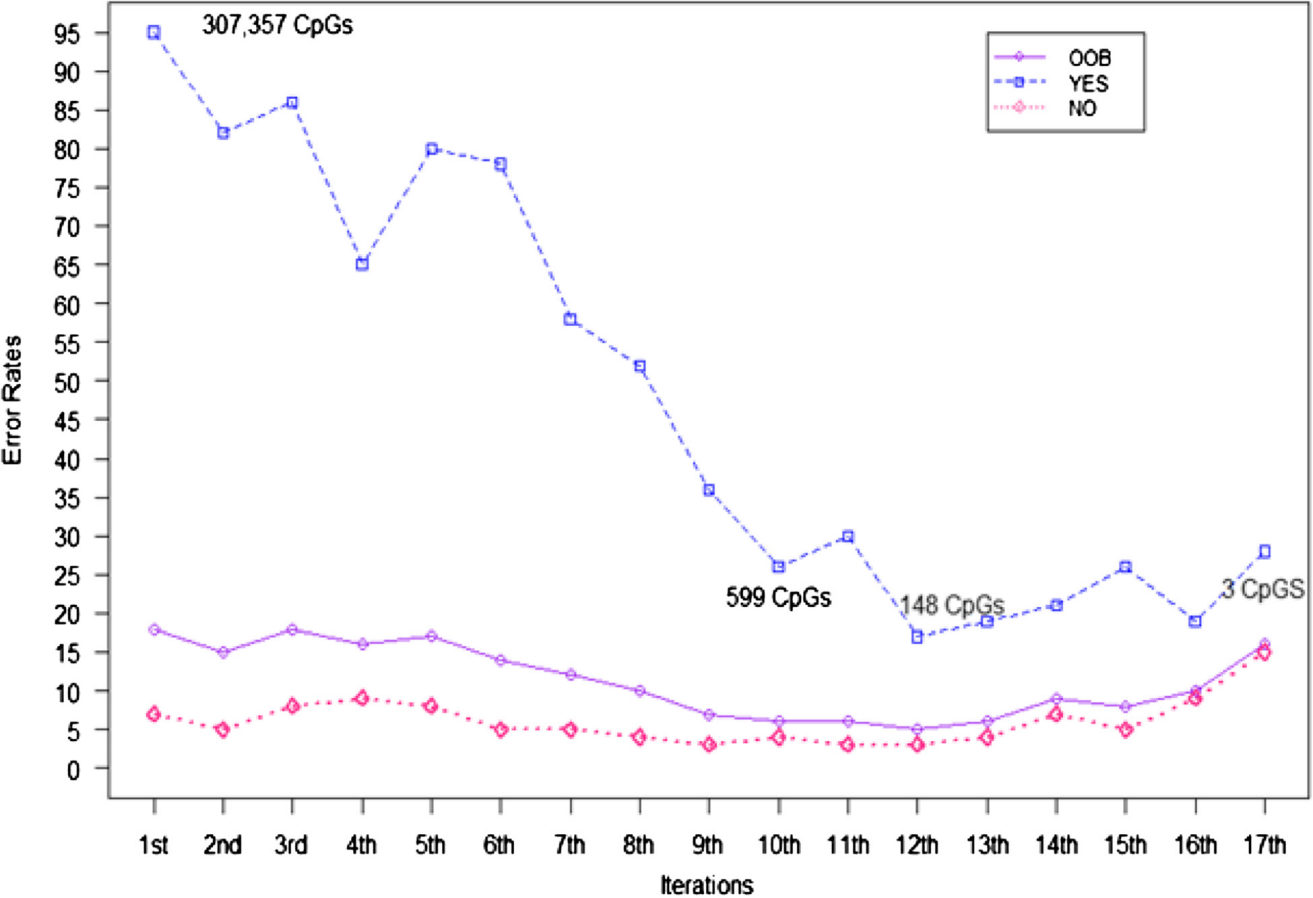
Misclassification error rates at each iteration of the recursive RF. *OOB* out of bag error rate (overall error), *YES* eczema, *No* non-eczema

In the screening process using recursive RF [14, 15], the parameters (*sampsize*, *mtry*, and *ntree*—details are in the “Statistical analysis” section) in the *randomForest*() R package were selected to achieve stabilized error rates. In total, pre-processed DNA methylation of 307,357 CpGs in the F_1_ generation was included in the screening. The results of the recursive RF (Table 2, Fig. 1; details in the “Statistical Analysis” section) Indicated that a total of 140 CpGs (after excluding 8 CpGs located on the X chromosome) passed the screening showing potential association with eczema. The exclusion of the 8 CpGs were due to the potential bias measurement of DNA-M for different genders. Nevertheless, in the following analyses (log-linear models below), we assessed whether gender played a role in the association of DNA-M with eczema.

**Table 2:** The performance of recursive RF at each iteration

Iteration	Number of CpGs	(OOB-ER) Overall misclassification	Eczema misclassification	Non-eczema misclassification
1	307,357	18.6 %	95.7 %	7.5 %
2	153,678	15.3 %	82.6 %	5.6 %
3	76,838	18.6 %	87.0 %	8.8 %
4	38,419	16.1 %	65.2 %	9.1 %
5	19,208	17.8 %	80.4 %	8.8 %
6	9604	14.2 %	78.3 %	5.0 %
7	4802	12.3 %	58.7 %	5.6 %
8	2401	10.7 %	52.2 %	4.7 %
9	1200	7.9 %	37.0 %	3.8 %
10	599	6.8 %	26.1 %	4.1 %
11	298	6.6 %	30.4 %	3.1 %
12^a^	148	5.2 %	17.4 %	3.4 %
13	74	6.3 %	19.6 %	4.4 %
14	37	9.3 %	21.7 %	7.5 %
15	18	8.5 %	26.1 %	5.9 %
16	9	10.7 %	19.6 %	9.4 %
17	3	16.9 %	28.2 %	15.3 %

*OOB-ER* out of bag error rate

^a^The 12th iteration had the lowest misclassification error rate

Further examination of these 140 CpGs from F_1_ using log-linear models indicated that 88 out of 140 CpGs had a statistically significant linear association with eczema at age 18 (FDR-adjusted *P* value <0.05) (Additional file 1: Table S1). We also tested the statistical significance of the interaction between DNA-M and gender; none of the FDR-adjusted *P* values were <0.05.

We assessed the biological pathways enriched within the genes annotated to those 140 CpGs using DAVID (Table 3). The most significantly enriched pathways related to the creation of transcriptional variety through genetic (e.g., polymorphism) and regulatory (e.g., alternative splicing) mechanisms. The remainder of the significantly enriched pathways included several pathways mechanistically linked to epithelial barrier integrity and cell adhesion, which are of key importance in eczema: examples include cadherins (protocadherin gamma, *P* = 1.8 × 10^−16^; cadherin 6 domain, *P* = 1.5 × 10^−10^; and cadherin N-terminal domain, *P* = 3.6 × 10^−10^), gap junctions (*P* = 2.6 × 10^−6^), cell adhesion (cell-cell adhesion, *P* = 2.7 × 10^−10^; cell adhesion, *P* = 2.2 × 10^−5^), tight junctions (*P* = 1.6 × 10^−5^), melanogenesis (*P* = 7.1 × 10^−5^), and apoptosis (*P* = 7.3 × 10^−3^).

**Table 3:** Terms significantly enriched in functional annotation and pathway analysis and genes present in the pathways potentially associated with eczema (FDR-adjusted *P* value; FDR = 0.05)

Term	FDR-adjusted *P* value	
Polymorphism	4.7 × 10^−145^	
Sequence variant	2.3 × 10^−111^	
Alternative splicing	6.8 × 10^−74^	
Splice variant	1.6 × 10^−46^	
Phosphoprotein	6.2 × 10^−25^	
Protocadherin gamma	1.8 × 10^−16^	*PCDHGA1*, *PCDHGA2*, *PCDHGA3*, *PCDHGA4*, *PCDHGA5*, *PCDHGA6*, *PCDHGA7*, *PCDHGA8*, *PCDHGA9*, *PCDHGB1*,
Disease mutation	4.9 × 10^−16^	*PCDHGB2*, *PCDHGB3*, *PCDHGB4*, *PCDHGB5*
Domain: cadherin 6	1.5 × 10^−10^	*FAT1*, *PCDHGA1*, *PCDHGA2*, *PCDHGA3*, *PCDHGA4*, *PCDHGA5*, *PCDHGA6*, *PCDHGA7*, *PCDHGA8*, *PCDHGA9*, *PCDHGB1*, *PCDHGB2*, *PCDHGB3*, *PCDHGB4*, *PCDHGB5*
Cadherin, N-terminal	3.6 × 10^−10^	*PCDHGA1*, *PCDHGA2*, *PCDHGA3*, *PCDHGA4*, *PCDHGA5*, *PCDHGA6*, *PCDHGA7*, *PCDHGA8*, *PCDHGA9*, *PCDHGB1*, *PCDHGB2*, *PCDHGB3*, *PCDHGB4*, *PCDHGB5*
Pathways in cancer	8.5 × 10^−8^	
Membrane	1.1 × 10^−7^	
Regulation of actin cytoskeleton	1.8 × 10^−7^	
Long-term depression	9.0 × 10^−7^	
Calcium ion binding	1.1 × 10^−6^	
Plasma membrane	2.2 × 10^−6^	
Glycoprotein	2.4 × 10^−6^	
Gap junction^a^	2.6 × 10^−6^	*GNAS*, *GNAI2*, *GNAI3*, *GUCY1A3*, *MAP2K1*, *PDGFA*,*PRKG1*
Cell-cell adhesion	2.7 × 10^−6^	*CD164*, *CLDN5*, *CDSN*,*DAB1*, *FAT1*, *FGF6*, *PARD3*, *PTPRF*, *PCDHGA1*, *PCDHGA2*, *PCDHGA3*, *PCDHGA4*, *PCDHGA5*, *PCDHGA6*, *PCDHGA7*, *PCDHGA8*, *PCDHGA9*, *PCDHGB1*,*PCDHGB2*,*PCDHGB3*, *PCDHGB4*, *PCDHGB5*
Homophilic cell adhesion	6.1 × 10^−6^	
Chemokine signaling pathway	1.0 × 10^−5^	
Focal adhesion	1.3 × 10^−5^	
Axon guidance	1.3 × 10^−5^	*CLDN5*, *GNAI2*, *GNAI3*, *CSNK2B*, *MAGI2*, *MYL12B*, *PARD3*
Tight junction^a^	1.6 × 10^−5^	
Biological adhesion	1.7 × 10^−5^	
Cell adhesion	2.2 × 10^−5^	*AEBP1*, *CD164*, *CD36*, *CLDN5*, *COL11A2*, *COL20A1*, *CDSN*, *DAB1*, *FAT1*, *FGF6*, *IGSF11*, *LAMA4*, *LAMC1*, *NELL2*, *NTM*, *PARD3*, *PTPRF*, *PPFIA1*, *PCDHGA1*, *PCDHGA2*, *PCDHGA3*, *PCDHGA4*, *PCDHGA5*, *PCDHGA6*, *PCDHGA7*, *PCDHGA8*, *PCDHGA9*, *PCDHGB1*, *PCDHGB2*, *PCDHGB3*, *PCDHGB4*, *PCDHGB5*
Coiled coil	2.6 × 10^−5^	
Melanogenesis^a^	7.1 × 10^−5^	*GNAS*, *CREB3*, *GNAI2*, *GNAI3*, *MAP2K1*, *WNT10B*
Vascular smooth muscle contraction	1.1 × 10^−4^	
Chromosomal rearrangement	2.6 × 10^−4^	
Cardiac muscle contraction	2.7 × 10^−4^	
Intracellular signaling cascade	4.5 × 10^−4^	
Cell membrane	4.7 × 10^−4^	
Cell fraction	4.8 × 10^−4^	
Prostate cancer	6.2 × 10^−4^	
Ion binding	6.8 × 10^−4^	
Acetylation	7.6 × 10^−4^	
Signal	8.3 × 10^−4^	
Transmembrane	1.0 × 10^−3^	
Mutagenesis site	1.1 × 10^−3^	
Cation binding	1.2 × 10^−3^	
Lysine degradation	1.4 × 10^−3^	
Leukocyte trans endothelial migration	1.5 × 10^−3^	
Lysosome	1.5 × 10^−3^	
Transcription factor binding	3.9 × 10^−3^	
Melanoma^a^	4.6 × 10^−3^	*E2F2*, *FGF6*, *MAP2K1*, *PDGFA*
Tumor suppressor	5.0 × 10^−3^	
Nucleotide binding	5.0 × 10^−3^	
Endocytosis	7.0 × 10^−3^	
Apoptosis^a^	7.3 × 10^−3^	*CHP2*, *NTRK1*, *PPP3CA*, *RIPK1*
Small cell lung cancer	7.3 × 10^−3^	
Nucleus	1.1 × 10^−2^	
Cell projection	1.7 × 10^−2^	
Positive regulation of cellular biosynthetic process	4.4 × 10^−2^	
Transcription co-activator activity	4.9 × 10^−2^	

^a^Represents pathways which are involved in eczema with their genes

### Replication results

**Fig. 2:**
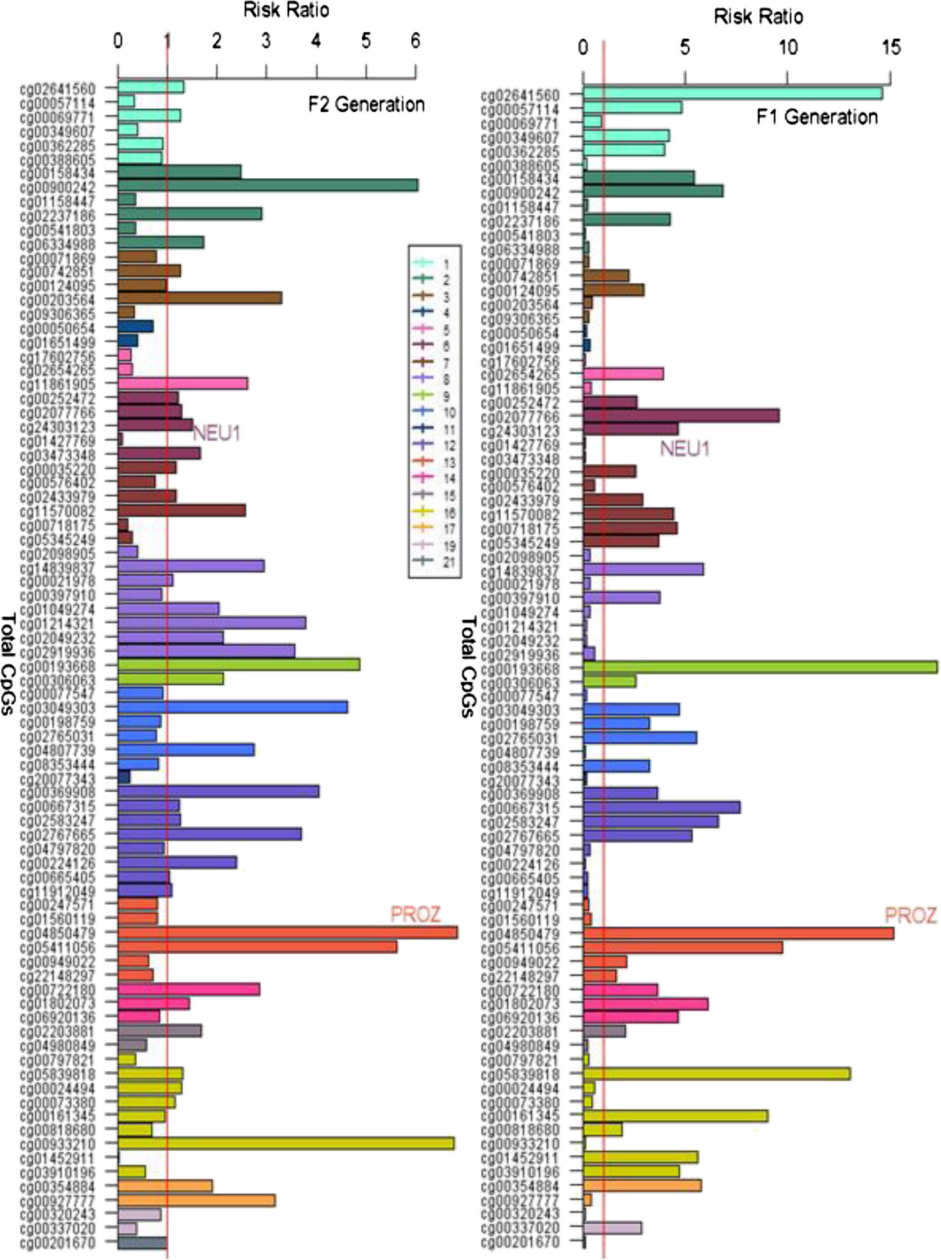
The risk ratios of 83 eczema-associated CpGs sorted by chromosome from 1 to 21. The *numbers* in the textbox are chromosome indices, which are represented by different colors in the bar graphs. The horizontal *red line* represents the risk ratio of one

We then replicated the findings from the F_1_ generation in the F_2_ generation. In total, 83 out of the 88 CpGs identified in the F_1_ were also present in the F_2_ dataset (the 5 CpG sites in the F2 were excluded after quality control). DNA methylation at 41 CpGs (out of 83) showed the same direction of changes with eczema in both the F_1_ and F_2_ generations (Table 4, Fig. 2). Of these 41 CpGs, two were statistically significantly associated with eczema risk in both generations (Table 4); cg04850479 in the *PROZ* gene showed adjusted risk ratio (RR) of 15.19 (95 % confidence interval (CI) 1.71 to 79.50) in the F_1_ and 6.82 (95 % CI 1.52 to 30.62) in the F_2_ and cg01427769 in the *NEU1* gene showed adjusted RR of 0.13 (95 % CI 0.03 to 0.46) in the F_1_ and 0.09 (95 % CI 0.03 to 0.36) in the F_2_. We further assessed the association of DNA methylation of these 2 CpGs with corresponding gene expressions in the F_2_ generation. No statistically significant associations were identified. Among the remaining CpGs not replicated in the F2 generation, about 60 % CpGs (*n* = 25 CpGs) showed a statistically significant difference in DNA methylation between the two generations (based on two sample two sided *t* tests) after adjusting for multiple testing. Since some of the F2 generation are offsprings of subjects in the F1 generation, the findings tend to be conservative. The above analyses were adjusted for estimated cell type proportions [16].

**Table 4:** The 41 CpGs that had the same direction of effect with eczema in both F_1_ and F_2_ generations based on log-linear models

CpGs	F_1_-Risk Ratio	95 % CI-F_1_	F_2_-risk ratio	95 % CI-F_2_	Gene
cg00193668	17.29	2.90, 102.87	4.86	0.89, 26.4	*HINT2*
cg04850479^a^	15.19	3.07, 75.17	6.82	1.52, 30.6	*PROZ*
cg02641560	14.50	3.39, 62.65	1.33	0.13, 12.8	*RCAN3*
cg05839818	13.02	2.34, 72.26	1.3	0.13, 12.1	
cg05411056	9.73	2.64, 35.81	5.61	1.44, 21.85	
cg02077766	9.60	2.14, 43.07	1.29	0.38, 4.33	*PTCRA*
cg00667315	7.66	1.88, 31.21	1.25	0.19, 8.0	
cg00900242	6.86	1.26, 37.20	6.04	0.75, 48.6	
cg02583247	6.61	2.05, 21.33	1.27	0.26, 6.10	*FGF6*
cg01802073	6.10	1.40, 26.43	1.43	0.24, 8.61	*CGRRF1*
cg14839837	5.90	1.63, 21.39	2.94	0.73, 11.7	*ARHGEF10*
cg00354884	5.77	1.95, 17.03	1.8	0.59, 6.03	*ABR*
cg00158434	5.43	1.75, 16.78	2.47	0.52, 11.5	*ALMS1P*
cg03049303	4.73	1.44, 15.57	4.61	0.77, 27.4	*C10orf76*
cg24303123	4.68	1.73, 12.65	1.49	0.50, 4.46	*RIPK1*
cg11570082	4.46	1.85, 10.71	2.56	0.58, 11.2	
cg02237186	4.26	1.24, 14.63	2.89	0.16, 51.1	*RRM2*
cg02654265	3.92	1.56, 9.87	0.29	0.05, 1.52	
cg00369908	3.65	1.34, 9.92	4.05	0.75, 21.6	*ING4*
cg00722180	3.64	1.22, 0.85	2.84	0.63, 12.7	*RBM25*
cg02433979	2.91	1.35, 6.27	1.17	0.37, 3.68	
cg00035220	2.62	1.19, 5.72	1.18	0.34, 4.03	*PTPRN2*
cg00252472	2.62	1.27, 5.40	1.22	0.44, 3.38	
cg00306063	2.59	1.12, 5.97	2.12	0.48, 9.19	*LOC100129066*
cg00742851	2.23	1.16, 4.28	1.26	0.45, 3.48	*LRRN1*
cg02203881	2.07	1.07, 4.00	1.67	0.46, 6.02	*PLA2G4D*
cg00576402	0.57	0.35, 0.92	0.76	0.28, 2.07	*PTPN12*
cg01560119	0.41	0.21, 0.80	0.79	0.37, 1.68	*SETDB2*
cg01651499	0.37	0.16, 0.85	0.41	0.12, 1.34	*GUCY1A3*
cg02098905	0.35	0.16, 0.76	0.41	0.14, 1.12	
cg04797820	0.33	0.17, 0.64	0.93	0.31, 2.76	*GLT1D1*
cg00247571	0.31	0.13, 0.75	0.89	0.26, 2.50	
cg00071869	0.30	0.13, 0.70	0.77	0.12, 4.88	*ATP1B3*
cg00797821	0.29	0.10, 0.82	0.36	0.06, 2.12	
cg01158447	0.24	0.09, 0.60	0.35	0.11, 1.14	*SLC40A1*
cg00077547	0.21	0.06, 0.70	0.91	0.25, 3.24	*TMEM26*
cg04980849	0.21	0.07, 0.60	0.57	0.16, 1.96	*LOC145663*; *GATM*
cg00050654	0.19	0.07, 0.51	0.71	0.20, 2.43	
cg20077343	0.19	0.06, 0.58	0.25	0.03, 1.83	*MUC6*
cg17602756	0.14	0.03, 0.64	0.26	0.05, 1.39	*SQSTM1*
cg01427769^a^	0.13	0.03, 0.46	0.09	0.02, 0.36	*NEU1*

^a^CpG sites significantly associated with eczema in both generations. For cg04850479, the *P* values are 0.0006 in the F_1_ generation and 0.0121 in the F_2_ generation, and for cg01427769, the *P* values are 0.0015 and 0.0007, respectively

## Discussion

This is the first study to explore epigenome-scale DNA methylation patterns associated with eczema. Using data from two generations, our study based on data of the F_1_ generation identified CpGs potentially associated with eczema status using the RF technique, which was further corroborated via log-linear models. In total, 140 CpGs were identified via RF, which were further assessed using log-linear models with 88 CpGs being statistically significantly associated with eczema risk after adjusting for cell type proportions and controlling for multiple testing. The remaining 52 CpGs were not corroborated in log-linear models. This is likely due to two reasons. Firstly, the 140 CpGs were identified based on their importance values in terms of minimizing misclassification errors other than statistical testing [11]. It is possible that the identified CpG sites did not have a statistically significant main effect on eczema risk. Secondly, among the 140 CpGs, complex non-linear interactions are likely to exist between multiple CpGs which may be difficult to parametrically identify using log-linear models. Using F_2_ generation data, around 50 % (41 CpGs) of these 88 CpGs identified in the F_1_ generation were further replicated. In particular, two CpGs showed statistically significant results in both F_1_ and F_2_: cg04850479 in the *PROZ* gene and cg01427769 in the *NEU1* gene. Although some studies have linked *NEU1* gene with asthma [17] via Th2-mediated airway inflammation [18, 17], and it is known that the Th2 pathway is also important for eczema [19, 20], based on our knowledge, no study has so far spotted its role in eczema. The insignificant findings on the association of DNA methylation of cg04850479 (in the *PROZ* gene) and cg01427769 (in the *NEU1* gene) is likely due to tissue-specific gene expression. That is, an early exposure has left a change in methylation in all tissues including blood but the gene is not expressed in blood but skin for eczema. It is also possible that the DNA methylation of these two CpGs is related to the production of dysfunctional transcripts.

Enrichment analysis of the CpG sites identified in the F_1_ generation highlighted pathways related to the creation of transcriptional variation and several biological pathways related to the epidermal barrier and involved in eczema (Table 3).

The skin barrier is crucial in maintaining skin integrity, and disruption of the epidermal barrier is one of the important mechanisms in the pathogenesis of eczema [21, 22]. Several studies reported that skin barrier dysfunction is a result of the impairment of tight junction function in eczema patients [23–26]. Cadherins and protocadherins are transmembrane proteins important for cell-to-cell adhesion and epithelial integrity and have been associated with eczema and asthma in genetic studies [27]. Chronic eczema and several other dermatoses are also related to hyperpigmentation of the skin [28]. Our study detected differentially methylated CpGs within genes in pathways relating to epidermal barrier integrity and eczema pathogenesis, including cadherins, gap junction, cell adhesion, tight junction, melanogenesis, and apoptosis (Table 3). Their biological functions suggest these eczema-associated CpGs are of special interest, and they are potential epigenetic biomarkers for eczema. The detection of eczema-associated differential methylation within pathways already known to be associated with eczema is reasonable and suggests that epigenetic and genetic variation may work together to regulate eczema-associated gene expression in the genes identified here, as has already been observed in other eczema-associated genes [22].

Several limitations were identified in the process of our study. Although the 140 CpGs were chosen based on the least misclassification error rate, it is possible that some CpGs were incorrectly removed and vice versa. Also, cord blood contains a small amount of maternal cells [29], which may bias the measure of DNA methylation, but our cell type correction performed in this study was expected to reduce the bias. Findings from the F_1_ generation were partially replicated in the F_2_ generation. This could be due to age playing a role in the CpGs predicting eczema; adolescence transition has the potential to revise DNA methylation. This is supported by our comparison of DNA methylation between the F1 and F2 generations among the CpGs not replicated. Not all CpGs selected by random forests were involved in known eczema-associated biological pathways, which may be due to complex interactions between the CpGs hence requires further investigation. It is possible that some of the identified CpGs may be associated with the severity of eczema. Hence, there is a need to further examine potential associations of DNA methylation of those CpG sites with eczema severity. For multiple CpG sites, DNA methylation was associated with eczema in the F_1_ generation at age 18. These CpG sites could be risks or consequences of eczema. However, CpGs replicated in the F_2_ generation were measured in cord blood before the onset of eczema and thus have the potential to predict eczema.

## Conclusions

This is the first epigenome-scale association study of eczema employing a classification technique (recursive RF), and we identified eczema-associated CpG sites. The findings added to the existing knowledge that recursive RF can be successfully employed in drawing actionable results from complex datasets. Genes annotated to eczema-associated CpGs were significantly enriched in pathways related to the creation of transcriptional variation and pathways relating to epidermal barrier function and eczema. Furthermore, the study identified for the first time that the *PROZ* and *NEU1* genes are potential predictors of eczema.

## Methods

### Isle of Wight birth cohort

The Isle of Wight (IoW) birth cohort was established to study the natural history of allergic diseases among children who were born between January 1, 1989 and February 28, 1990 on the Isle of Wight, UK. The study was approved by the local research ethics committee and written informed consent was obtained from the parents. After exclusion of adoptions, perinatal deaths, and refusal, 1456 children (95 %) were enrolled. Children were followed-up at ages 1 (*n* = 1167), 2 (*n* = 1174), 4 (*n* = 1218), 10 (*n* = 1373), and 18 years (*n* = 1313); detailed questionnaires were administered at each follow-up. Details of the birth cohort have been described elsewhere [4, 30, 31]. A total of 244 women and 122 men at age 18 years were randomly selected from the cohort for epigenome-scale DNA methylation studies. Ethics approvals were obtained from the Isle of Wight Local Research Ethics Committee (now named the National Research Ethics Service, NRES Committee South Central – Southampton B) at recruitment and for the subsequent follow-ups (06/Q1701/34).

### Outcome: eczema phenotype data collection

Eczema was defined as chronic or chronically relapsing itchy dermatitis lasting more than 6 weeks with characteristic morphology and distribution [32], following Hanifin and Rajka criteria [5].

### DNA methylation

DNA was extracted from whole blood and umbilical cord blood using a standard salting out procedure [33]. DNA concentration was determined by Qubit quantitation. One microgram of DNA was bisulfite-treated using the EZ 96-DNA methylation kit (Zymo Research, Irvine, CA, USA) following the manufacturer’s standard protocol.

Epigenome-scale DNA methylation was assessed using the Illumina Infinium HumanMethylation450 Beadchip (Illumina, Inc., San Diego, CA, USA), which interrogates >484,000 CpGs associated with approximately 24,000 genes. Arrays were processed using a standard protocol as described elsewhere [7], with multiple identical control samples assigned to each batch to assess assay variability, and samples were randomly distributed on microarrays to control against batch effects. The methylation level (*β* value) for each CpG was determined using the Methylation module of GenomeStudio software (Illumina, Version 2011.1).

Methylation levels for each CpG site are recorded as beta (*β*) values, which represent the proportion of methylated (*M*) over methylated (*M*) plus unmethylated (*U*) probes (*β* = *M*/[*c* + *M* + *U*], with constant *c* introduced for the situation of too small *M* + *U*) and can be interpreted as percentage methylation. These values were utilized in the RF screening process described below; however, *β* values close to 0 or 1 tend to suffer from severe heteroscedasticity; therefore, logit-transformed *β* values (*M* values, approximated by log_2_(β / (1-β)) [34] were used in other analyses including log-linear models.

### Pre-processing DNA methylation data

In our study, the detection *P* value reported by GenomeStudio was used as a QC measure of probe performance. Probes whose detection *P* values were >0.01 in >10 % of the samples were removed [35]. Methylation data were then pre-processed using the Bioconductor IMA (Illumina methylation analyzer) package and ComBat was used to perform peak correction and adjust for inter-array variation [36, 37]. To ensure that our findings were not biased by SNPs affecting measurement of methylation levels, we excluded all probes with a potential SNP in the probe sequence. After pre-processing, a total of 307,357 CpGs were retained in the DNA methylation dataset.

### Statistical analysis

Pearson’s *χ*^2^ tests were used to determine if prevalence of eczema differed between the sexes. *P* values were considered significant at a level of 0.05. To make sure that our findings are not a result of confounding due to cell types, we ran the analyses by adjusting for estimated proportions of CD8^+^ T cells, CD4^+^ T cells, natural killer cells, B cells, monocytes, and granulocytes. Cell type proportions were estimated as described previously [16].

The random forest package, *randomForest*(), in R was utilized to conduct the recursive RF analyses [38, 15, 14]. The parameter *sampsize* refers to the size of the sample of training data sets that is to be obtained for classification. The number of variables that are randomly sampled as predictors at each split is called *mtry*, whereas, *ntree* is a parameter referring to the total number of trees that are to be grown in the forest. In order to improve the prediction accuracy of the RF algorithm, these three parameters were repeatedly altered until the lowest misclassification rate was obtained. We decided whether to use a balanced *sampsize* of equal eczema and non-eczema cases such as 20 eczema and 20 non-eczema cases or 30/30 or 40/40. We also studied imbalanced RFs with *sampsize* such as 46/320 or 20/40 for the training sets by using the default values for *mtry and ntree*. We then tested the prediction accuracy of the RFs at different combinations of *mtry* (√*p*, 2*√*p*, 0.1*p*, 0.15*p*, 0.2*p*, and 0.25*p*) where *p* is number of variables and *ntree* (200, 500, 1000, and 1500). Once the optimal parameter values were selected, the recursive RF algorithm was implemented. Mean Decrease Gini (MDG) served as a variable importance measure (VIM) for our study as it was shown to be more robust in previous research [39].

DNA methylation at 307,357 CpGs along with sex and eczema status in the F_1_ generation served as input in *randomForest*(), and the CpGs were subjected to data reduction, repeatedly dropping 50 % of variables with the lowest VIMs until the misclassification rate showed a significant increase.

After testing for *sampsize* (both equal and unequal) with different combinations (both with and without eczema), we set *sampsize* = (31, 31), *mtry* = 0.2*p* (where *p* is the available number of variables) and *ntree* = 500. We applied RF to pre-processed DNA methylation data containing 307,357 CpGs in the F_1_ generation, ran a total of 17 iterations, and at each iteration, recorded the misclassification rate (Table 2, Fig. 1). The lowest overall misclassification error rate (of eczema and eczema-free) was 5.2 %, with a corresponding least misclassification rate of 17.4 % for eczema at the 12th iteration. The overall misclassification rate dropped from 18.6 % in the first iteration to 5.2 % in the 12th iteration, and the eczema misclassification error rate dropped to 17.4 % at the end of 12th iteration from 95.7 % in the first iteration.

The CpGs identified from the recursive RF [40] were assessed for enrichment of biological pathways using DAVID [12] bioinformatics tool and examined for their association with eczema at age of 18 years by use of log-linear models. Multiple testing was adjusted by controlling false discovery rate of 0.05 in the pathway analysis and log-linear models. Since differential cell types in the peripheral blood are known to have confounding effect on the final result [16], we adjusted for cell type correction. For genes of particular interest (e.g., showing statistical significance in both generations in log-linear models), robust regressions are applied to assess the association of DNA methylation and corresponding gene expressions in the F_2_ generation. For this last test, multiple testing is adjusted within genes based on the number of CpG sites available of that gene.

### Replication cohort

**Fig. 3:**
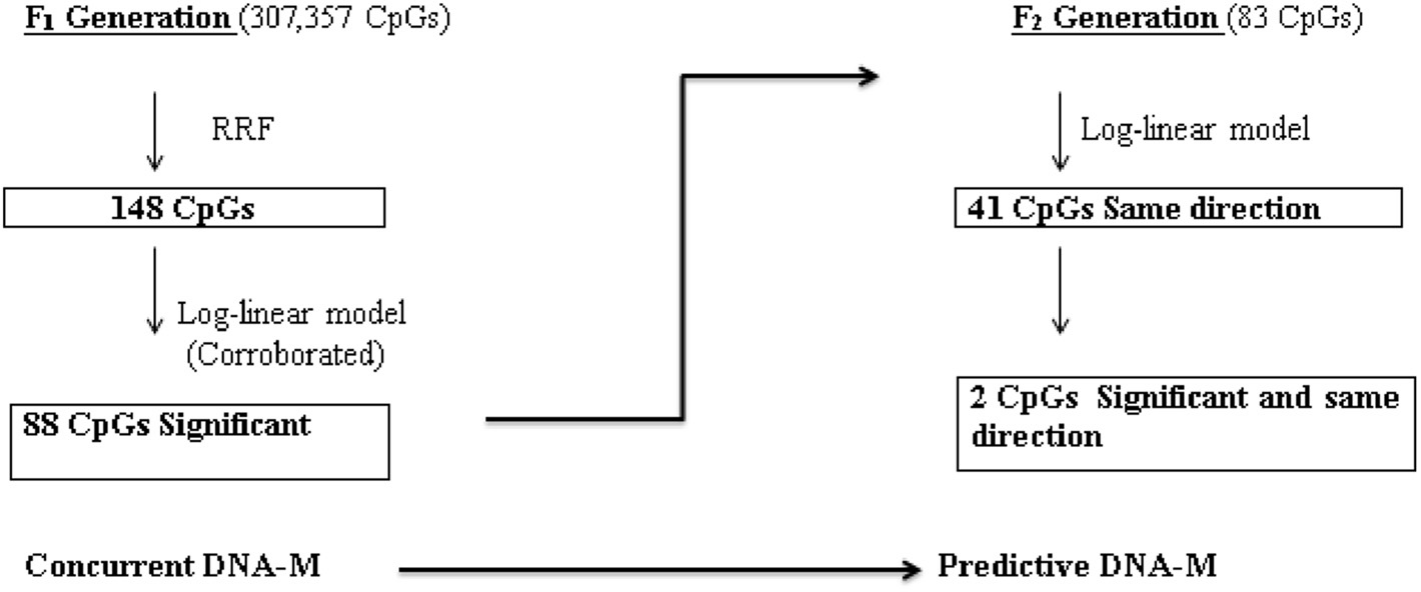
Flow chart of statistical analyses and the number of CpG sites after each analysis in the F_1_ and F_2_ generations. RRF: recursive random forest

The IoW F_2_ generation cohort includes the offspring of the IoW 1989 birth cohort. In the F_2_ generation, repeated measures of eczema at ages 3, 6, and 12 months were recorded in a sample of *n* = 116 children. DNA methylation was measured in umbilical cord blood. To replicate the findings from the F_1_ generation, log-linear models with repeated measures of eczema were used in F_2_ generation analyses. Figure 3 represents the summary of statistical analysis and sample size for each analysis conducted in this study.

## Additional file

Additional file 1: Table S1. Beta coefficients and *P* values after log-linear model of the 140 CpGs present in the 12th iteration of the RF algorithm output and their genetic details. Software necessary to view: Adobe Reader.

## Abbreviations

CpG: cytosine-phosphate-guanine
DNA-M: DNA methylation
IoW: Isle of Wight
RF: random forest
RR: risk ratio
