# Supplementary material for: Identifying CpG sites associated with eczema via random forest screening of epigenome-scale DNA methylation
Source: Clin Epigenetics. 2015 Jul 21;7(1):68. doi: 10.1186/s13148-015-0108-y (PMC4508804; doi:10.1186/s13148-015-0108-y)
Supplement: Additional file 1: Table S1. — Beta coefficients and P values after log-linear model of the 140 CpGs present in the 12th iteration of the RF algorithm output and their genetic details. Software necessary to view: Adobe Reader. [file 13148_2015_108_MOESM1_ESM.pdf]

Supplementary Table 1. Beta coefficients and *P* values after log-linear model of the 140 CpGs present in the 12th iteration of the RF algorithm output and their Genetic details

CpGs	CHR$	UCSC* chromosomal location	Gene	UCSC* Gene Region	USCS* CpG Island Relation	β-coefficients	P-Value
**cg00057114**	1	6344362	*ACOT7*	Body	S_Shelf	1.57	0.032
**cg00069771**	1	233089275	*C1orf57*	Body	S_Shelf	-0.11	0.031
**cg00349607**	1	39546688	*MACF1*	TSS1500	N_Shore	1.44	0.026
**cg00362285**	1	236444212	*ERO1LB*	Body	N_Shore	1.38	0.026
**cg00388605**	1	89150448	*PKN2*	Body	Island	-1.55	0.028
**cg02641560**	1	24828811	*RCAN3*	TSS1500	N_Shore	2.68	0.011
**cg00158434**	2	73871782	*ALMS1P*	TSS1500		1.69	0.017
**cg00541803**	2	120301835	*PCDP1*	TSS1500	Island	-2.02	0.031
**cg00900242**	2	200929967				1.92	0.043
**cg01158447**	2	190445074	*SLC40A1*	Body	N_Shore	-1.40	0.016
**cg02237186**	2	10263043	*RRM2;RRM2*	Body	Island	1.45	0.039
**cg06334988**	2	220095083	*ANKZF1;ATG9A*	Body; TSS1500	S_Shore	-1.12	0.009
**cg00071869**	3	141599902	*ATP1B3*	Body	S_Shelf	-1.17	0.023
**cg00124095**	3	72305082				1.10	0.016
**cg00203564**	3	176133845				-0.80	0.038
**cg00742851**	3	3843342	*LRRN1*	5'UTR	S_Shore	0.80	0.034
**cg09306365**	3	61547944	*PTPRG*	1stExon; 5'UTR	Island	-1.17	0.047
**cg00050654**	4	4576493			N_Shore	-1.64	0.014
**cg01214900**	4	66784395				0.84	0.104
**cg01651499**	4	156646379	*GUCY1A3*	Body		-0.99	0.038
**cg02654265**	5	137086868			N_Shelf	1.36	0.017
**cg11861905**	5	176045983			N_Shore	-0.89	0.038
**cg17602756**	5	179246001	*SQSTM1*	5'UTR	N_Shore	-1.92	0.028
**cg00252472**	6	150739173				0.96	0.026
**cg02077766**	6	42891866	*PTCRA*	Body	N_Shore	2.26	0.017
**cg03473348**	6	33160976	*COL11A2*	TSS1500	Island	-1.84	0.017
**cg24303123**	6	3076314	*RIPK1*	TSS1500		1.54	0.016
**cg00035220**	7	158197980	*PTPRN2*	Body	N_Shore	0.96	0.034
**cg00576402**	7	77167212	*PTPN12*	TSS200; Body	Island	-0.55	0.042
**cg00718175**	7	158807362	*LOC154822*	Body		1.53	0.043
**cg02433979**	7	128824829			N_Shelf	1.07	0.024
**cg05345249**	7	80291832	*CD36*	Body		1.30	0.016
**cg11570082**	7	116899446				1.49	0.014
**cg00021978**	8	105476917	*DPYS*	Body	N_Shore	-1.06	0.042
**cg00397910**	8	80522577	*STMN2*	TSS1500	N_Shore	1.33	0.016
**cg01049274**	8	146228340	*ZNF252;C8orf77*	TSS200; Body	Island	-1.02	0.046
**cg01214321**	8	26435429	*DPYSL2*	5'UTR; 1stExon	Island	-1.79	0.038

**cg02049232**	8	145556129	*SCRT1*	3'UTR	Island	-1.65	0.024
**cg02098905**	8	119759090				-1.02	0.026
**cg02919936**	8	70982285	*PRDM14*	5'UTR	Island	-0.57	0.038
**cg14839837**	8	1834174	*ARHGEF10*	Body	S_Shelf	1.77	0.025
**cg00193668**	9	35814283	*HINT2*	Body	N_Shore	2.85	0.016
**cg00306063**	9	92289643	*LOC100129066*	Body	N_Shore	0.95	0.043
**cg00077547**	10	63170372	*TMEM26*	Body		-1.55	0.03
**cg00198759**	10	81444321	*LOC650623*	Body	Island	1.18	0.047
**cg02765031**	10	85462980				1.71	0.009
**cg03049303**	10	103816154	*C10orf76*	TSS1500	S_Shore	1.55	0.028
**cg04807739**	10	88779914	*FAM25A*	TSS200		-2.03	0.024
**cg08353444**	10	60506653	*BICC1*	Body		1.18	0.026
**cg20077343**	11	1036307	*MUC6*	Body	N_Shore	-1.62	0.017
**cg00224126**	12	62861053	*MON2*	1stExon	S_Shore	-2.18	0.024
**cg00369908**	12	6772357	*ING4*	TSS200	Island	1.29	0.028
**cg00665405**	12	50899548	*DIP2B*	Body	S_Shore	-1.45	0.022
**cg00667315**	12	295951			N_Shelf	2.03	0.021
**cg02583247**	12	4555311	*FGF6*	TSS1500	S_Shore	1.88	0.016
**cg02767665**	12	129788385	*TMEM132D*	Body	S_Shore	1.67	0.016
**cg04797820**	12	129460559	*GLT1D1*	Body		-1.09	0.016
**cg11912049**	12	50361401			Island	-1.38	0.016
**cg00949022**	13	73356224	*PIBF1;DIS3*	TSS200; 1stExon; 5'UTR	Island	0.77	0.038
**cg01560119**	13	50025780	*SETDB2*	5'UTR; 1stExon		-0.87	0.026
**cg04850479**	13	113811569	*PROZ*	TSS1500	S_Shelf	2.72	0.014
**cg05411056**	13	112852819			S_Shelf	2.27	0.014
**cg22148297**	13	99910587	*GPR18;UBAC2*	1stExon; Body; 5'UTR		0.49	0.037
**cg00722180**	14	73525139	*RBM25*	TSS200	Island	1.29	0.038
**cg01802073**	14	54976343	*CGRRF1*	TSS1500	Island	1.80	0.034
**cg06920136**	14	21945858	*RAB2B;TOX4*	TSS1500; Body	S_Shore	1.54	0.026
**cg00229754**	15	25018302			Island	-0.55	0.149
**cg02203881**	15	42386909	*PLA2G4D*	TSS200		0.73	0.047
**cg04980849**	15	45671347	*LOC145663;GATM*	Body; TSS1500	S_Shore	-1.53	0.017
**cg00024494**	16	73096842			N_Shore	-0.50	0.041
**cg00073380**	16	5122085	*ALG1*	Body	Island	-0.78	0.038
**cg00161345**	16	66866226	*NAE1*	TSS1500	S_Shore	2.19	0.014
**cg00797821**	16	49888944			Island	-1.20	0.038
**cg00818680**	16	68881254	*TMCO7*	Body	S_Shelf	0.65	0.026
**cg00933210**	16	3233117			Island	-2.13	0.045
**cg01452911**	16	86512095				1.72	0.016
**cg03910196**	16	2256384	*MLST8*	Body	S_Shore	1.55	0.031
**cg05839818**	16	85634031				2.56	0.017
**cg00354884**	17	1057541	*ABR*	Body; 5'UTR		1.75	0.016

**cg00927777**	17	1960199	*HIC1*	Body	Island	-0.92	0.026
**cg00320243**	19	18314691	*RAB3A*	5'UTR	Island	-1.91	0.038
**cg00337020**	19	2241089	*SF3A2*	5'UTR	S_Shelf	1.04	0.028
**cg00201670**	21	46221900	*UBE2G2*	TSS200	Island	-1.95	0.043
**cg00015530**	8	145052950	*PARP10*	Body	S_Shore	0.32	0.244
**cg00019877**	9	115773738	*LOC169834*	5'UTR	N_Shore	1.82	0.024
**cg00546248**	1	2412743	*PLCH2*	Body	S_Shore	-0.45	0.186
**cg00778190**	1	95068029				0.54	0.101
**cg01059116**	1	54163070	*GLIS1*	5'UTR		0.31	0.048
**cg00164941**	2	73523056			S_Shelf	-0.59	0.11
**cg00729049**	2	171679402	*GAD1*	Body	Island	-1.10	0.031
**cg00405769**	4	187539852	*FAT1*	Body		-0.57	0.075
**cg00866215**	4	54956702			N_Shore	0.64	0.123
**cg05684300**	4	102267366	*PPP3CA*	Body	N_Shore	0.94	0.061
**cg00099094**	5	2334514				-0.23	0.189
**cg00106739**	5	177657311	*AGXT2L2*	Body	N_Shore	0.97	0.068
**cg00580497**	5	1153746			N_Shore	0.81	0.122
**cg00043444**	6	134384706				-0.23	0.633
**cg00155619**	6	33173278	*HSD17B8*	Body	S_Shore	0.97	0.08
**cg00491418**	6	112575482	*LAMA4*	5'UTR	Island	-1.72	0.066
**cg01427769**	6	31830600	*NEU1*	5'UTR; 1stExon	Island	-2.0	0.016
**cg12427444**	6	31923064	*RDBP*	Body	N_Shelf	-0.49	0.497
**cg00036614**	7	47093842			S_Shore	0.33	0.378
**cg00064840**	7	4784485	*FOXK1*	Body	N_Shore	1.42	0.068
**cg00106345**	7	27138396			S_Shore	-0.39	0.213
**cg00129269**	7	127228222	*ARF5*	TSS1500	Island	-0.92	0.068
**cg00301256**	7	82653693	*PCLO;PCLO*	Body		-0.57	0.264
**cg02474076**	7	127223255	*GCC1*	Body	N_Shore	1.47	0.126
**cg00161683**	8	95563352	*KIAA1429*	Body	N_Shelf	1.89	0.027
**cg00166750**	8	102138594			Island	-1.21	0.06
**cg00328935**	8	142517474	*FLJ43860*	TSS200		-0.34	0.359
**cg00405554**	8	65490897	*LOC401463*	TSS1500	N_Shore	1.34	0.084
**cg00449899**	8	53626436	*RB1CC1*	5'UTR	Island	-1.34	0.039
**cg00592695**	8	134545685	*ST3GAL1*	5'UTR		-0.92	0.094
**cg00598335**	8	11627530	*NEIL2*	5'UTR; 1stExon	Island	0.43	0.489
**cg00661018**	8	145769086	*KIAA1688*	Body	N_Shore	1.90	0.053
**cg00556719**	9	108457484	*TMEM38B*	Body	Island	1.98	0.075
**cg00152946**	10	130338781			Island	-1.53	0.068
**cg00338996**	11	45828080	*SLC35C1*	Body	S_Shore	0.75	0.06
**cg00478064**	11	118271813	*ATP5L*	TSS1500	N_Shore	1.61	0.09
**cg12079699**	11	131564481	*NTM*	Body	S_Shelf	-0.77	0.101
**cg00104348**	12	10772424	*STYK1*	3'UTR		-0.70	0.149
**cg00216961**	12	6054731	*ANO2*	Body		-0.91	0.189

**cg00240719**	12	132169130			N_Shore	-0.71	0.121
**cg00216180**	13	114778713	*RASA3*	Body	Island	0.88	0.213
**cg00247571**	13	50707065			N_Shore	-1.15	0.027
**cg01729020**	14	55035889	*SAMD4A*	Body	S_Shore	0.90	0.25
**cg00376910**	15	89765061	*RLBP1*	TSS200		-1.12	0.054
**cg00153543**	16	84259830	*KCNG4*	Body	S_Shelf	0.54	0.219
**cg00261912**	16	89777763	*C16orf7*	Body	Island	0.47	0.054
**cg00489954**	16	28855387	*TUFM*	Body	N_Shore	0.001	0.999
**cg00701514**	16	1183462			S_Shelf	-0.84	0.075
**cg00911376**	16	84274417	*KCNG4*	TSS1500	S_Shelf	0.67	0.162
**cg02455706**	16	84918851	*CRISPLD2*	Body		-0.78	0.032
**cg00416882**	18	44495671	*PIAS2*	Body	N_Shore	0.88	0.075
**cg00417323**	18	29307959			S_Shelf	0.76	0.093
**cg00486358**	18	54309442			S_Shelf	0.54	0.178
**cg01080902**	19	44039870	*ZNF575*	3'UTR	S_Shore	1.19	0.06
**cg00397859**	20	13768987	*C20orf7*	Body	S_Shelf	0.67	0.065
**cg00399938**	22	20118646	*ZDHHC8*	TSS1500	Island	0.44	0.25
**cg01133262**	22	38863905	*KDELR3*	TSS200	Island	0.87	0.226

CHR$ = chromosome number
UCSC*= University of South Carolina
